# Nematicidal Activity of *Stevia rebaudiana* (Bertoni) Assisted by Phytochemical Analysis

**DOI:** 10.3390/toxins12050319

**Published:** 2020-05-12

**Authors:** Nikoletta Ntalli, Konstantinos M. Kasiotis, Eirini Baira, Christos L. Stamatis, Kyriaki Machera

**Affiliations:** 1 Laboratory of Biological Control of Pesticides, Department of Pesticides Control and Phytopharmacy, Benaki Phytopathological Institute, 8 St. Delta Street, 14561 Athens, Greece; 2 Laboratory of Pesticides’ Toxicology, Department of Pesticides Control and Phytopharmacy, Benaki Phytopathological Institute, 8 St. Delta Street, 14561 Athens, Greece; k.kasiotis@bpi.gr (K.M.K.); e.baira@bpi.gr (E.B.); k.machera@bpi.gr (K.M.); 3 Stevia Hellas Coop, 6th klm Lamia-Karpenisi, PS 35131 Lamia, Greece; christos.stamatis@stevianet.gr

**Keywords:** *Meloidogyne incognita*, *Meloidogyne javanica*, phenolics, flavonoids, terpenes, bioactivity

## Abstract

To date, there has been great demand for ecofriendly nematicides with beneficial properties to the nematode hosting plants. Great efforts are made towards the chemical characterization of botanical extracts exhibiting nematicidal activity against *Meloidogyne spp.*, but only a small percentage of these data are actually used by the chemical industry in order to develop new formulates. On the other hand, the ready to use farmer produced water extracts based on edible plants could be a sustainable and economic solution for low income countries. Herein, we evaluate the nematicidal potential of *Stevia rebaudiana* grown in Greece against *Meloidogyne incognita* and *Meloidogyne javanica*, two most notorious phytoparasitic nematode species causing great losses in tomato cultivation worldwide. In an effort to recycle the plant’s remnants, after leaves selection for commercial use, we use both leaves and wooden stems to test for activity. In vitro tests demonstrate significant paralysis activity of both plant parts’ water extracts against the second-stage juvenile (J2) of the parasites; while, in vivo bioassays demonstrated the substantial efficacy of leaves’ powder (95% at 1 g kg^−1^) followed by stems. Interestingly, the incorporation of up to 50 g powder/kg of soil is not phytotoxic, which demonstrates the ability to elevate the applied concentration of the nematicidal stevia powder under high inoculum level. Last but not least, the chemical composition analyses using cutting edge analytical methodologies, demonstrated amongst components molecules of already proven nematicidal activity, was exemplified by several flavonoids and essential oil components. Interestingly, and to our knowledge, for the flavonoids, morin and robinin, the anthocyanidin, keracyanin, and a napthalen-2-ol derivative is their first report in Stevia species.

## 1. Introduction

**Figure 1:**
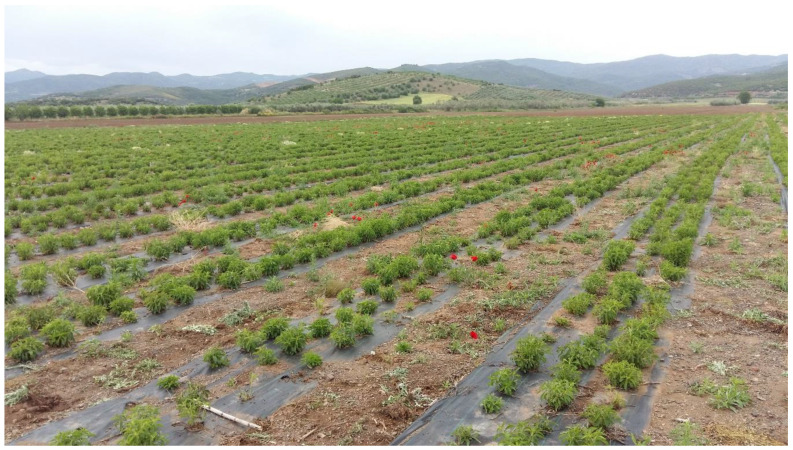
*Stevia rebaudiana* in cultivation at the Stevia Hellas Coop, 6th klm Lamia-Karpenisi, PS 35131, Lamia, Greece (composite photographs).

*Stevia rebaudiana* (Bertoni) Bertoni (Asteraceae) is mostly known for its contents in vastly sweet ent-kaurane (steviol) diterpene glycosides, natural non-caloric sweeteners, in use in Japan since the mid-1970s for sweetening uses [1]. *Stevia rebaudiana* is internationally known as “Sweet Herb”, and as “Caá hê-é” or “Kaá hê-é” and its native country is Paraguay [2,3,4,5]. The first reported ethnobotanical information regarding the presence of the sweet-tasting plant *S. rebaudiana* was made by Bertoni in 1905 and the second followed in 1918 [6,7] and at the beginning the leaves of the plant were used to sweeten maté and tereré drinks, infusion in hot or cold water, respectively [5]. *Stevia rebaudiana* is cultivated in Taiwan, China, Thailand, Korea, Malaysia, Brazil, Hawaii, Canada, and California [8]. In Greece, a pivotal cultivation region is located in the prefecture of Lamia, Central Greece (see Figure 1), where 57 farmers, members of the Agricultural Cooperative Stevia Hellas, are cultivating organic *S. rebaudiana* and export throughout Europe.

To date, *S. rebaudiana* and its glycosides are becoming more widespread both in the food industry and in the world of science. Specifically, *S. rebaudiana* preparations have been proven to exhibit various medical properties, like anti-inflammatory, chemo preventive effects, oral health-promoting, anticancer, antihypertensive, anti-hyperglycemic, antimicrobial, antihypertensive, anti-inflammatory, antioxidant, and immune-modulatory [9,10,11], while its refined extracts have GRAS (“Generally Regarded As Safe”) status in the U.S [12]. In the same context, previously major components of *S. rebaudiana’s* essential oil [13,14], such as spathulenol, caryophyllene oxide, manool oxide, *trans*-nerolinol, and α-cadinol are constituents of numerous natural products, with pronounced biological activities. Recently, plant protection properties of *S. rebaudiana* have been published against the phytopathogenic fungi *Fusarium oxysporum* [15,16] in the frame of developing safe and effective strategies, but alternative to the synthetic, plant protection products. In recent years there is an uprising global need for ecofriendly substitutes to the synthetic pesticides since many chemical groups of formulates have been associated with ecotoxicity concerns and, as a result, have been withdrawn from the market [17,18]. In the frame of discovering bioactive plant secondary metabolites, many are the references on the pesticidal properties of Asteraceae species [19,20] and, in particular, as regards the control of the root knot nematodes, a most harmful agricultural pest damaging crops worldwide [21]. *Meloidogyne* spp. are obligate endoparasites of plant roots and their reproduction depends on the induction of feeding sites on the host. For some greenhouse cultures, like zucchini, *M. incognita* and *M. javanica* have similar thermal needs to complete their life cycle, while their pathogenic potential (ability to cause disease) might differ in cases [22]. The genus *Meloidogyne* spp. is extremely polyphagous, parasitizing more than 3000 host plants and causing over $100 billion in annual crop losses worldwide [23].

*S. rebaudiana* is rich in plant secondary metabolites of biological activity [24,25] and it could potentially be an alternative nematode control source either through its harvested plant parts or by the culture remnants, which is the non-commercial plant parts, in the frame of the European Waste Framework Directive [26]. In addition, *Stevia rebaudiana* being an edible plant its un-fractionated and unpurified, crude water extracts could be developed into “basic substances” that is “nematicidal recipes” prepared by the farmer with low risk of harmfulness for soil, water, air, plants or animals [27].

To the best of our acquaintance this is the first report of *Stevia rebaudiana* as a nematicidal agent against *Meloidogyne* spp., along with the chemical composition analysis of the nematicidal extracts. In this context, we investigated: (a) the nematicidal potential of *S. rebaudiana* water extracts against two root knot nematodes species, that is *M. incognita* and *M. javanica*, in terms of J2 paralysis, (b) the efficacy of the powdered *S. rebaudiana* culture remnants against *M. incognita* and the secondary effects on tomato plants’ growth, and (c) the chemical composition of *S. rebaudiana* extracts in terms of targeted secondary metabolites (e.g glycosides and terpenes) and untargeted metabolomics analysis.

## 2. Results

### 2.1. Soil Amending with S. rebaudiana Leaves Powder (LP) and Wood Powder (WP) to Treat against M. incognita and Subsequent Biofertilization in Tomato Plants: A Dose-Response

**Figure 2:**
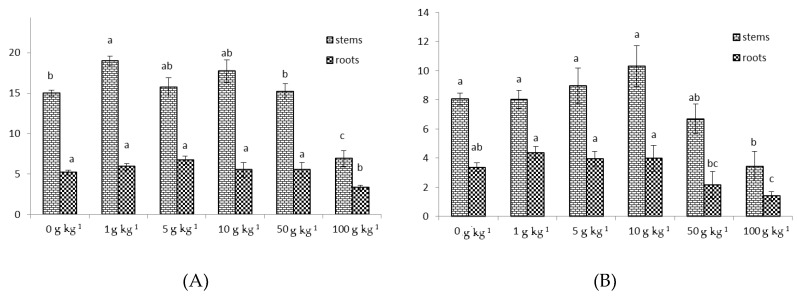
Tomato stems and root weights (g), as assessed after treatment with (**A**) leaves powder (LP) and (**B**) wood powder (WP) for *M. incognita* control in pot bioassays 40 days post experiment establishment. The data are means of five replicates with standard deviations. The means which are followed by the same letter are not significantly different according to Duncan test (*p* ≤ 0.05). Within each graph letters correspond to statistical differences amongst same pattern bars.

The LP exhibited best activity, since the EC_50_ value is to be lower that the smallest test concentration used in the tested dose range (Table 1). Specifically, according to nematode female counts per g of root, the nematicidal activity of LP tested at 1 and 5 g kg^−1^ was 60% and 95%, respectively (data not shown). The efficacy of NemGuard at the recommended dose 2 mg kg^−1^ was 95%. On the other hand, a clear dose response relationship was established for WP at the dose range of 1 to 100 g kg^−1^ soil, and the EC_50_ value was calculated at 3.13 g kg^−1^. Just to show infestation levels under our experimental conditions, we report that the counts of females per g of root tissue in control treatments were 120 ± 7. Under no circumstances was phytotoxicity observed at test concentrations, up to 50 g kg^−1^ soil, for both LP and WP (Figure 2). Only stems and roots weights of tomato plants treated with stevia powder at 100 g kg^−1^ soil were found significantly lower when considering control values.

### 2.2. Paralysis Effect of Leaves Water Extract (LWE) and Wood Water Extract (WWE) on the Plant Parasitic Nematode *M. incognita* and *M. javanica* Second Stage Juveniles (J2)

In general, clear dose and time response relationships were established for both LWE and WWE at the dose range of 5.4 to 0.042 mg mL^−1^ (Table 2). Only in the last assessment date was motility regained to a small extent for *M. javanica* immersed in LWE as well as for *M. incognita* and *M. javanica* J2 immersed in WWE. Nonetheless, paralysis exhibited at 48h remained constant thereafter, since juveniles moved in plain water never regained activity. Interestingly, the WWE was slower in paralyzing J2, since I h post experiment establishment no paralysis was evident, but, in the assessments that followed, similar EC_50_ values were established for both extract and nematode species.

### 2.3. Chemical Composition Analyses of LWE and EO; S. rebaudiana Glycosides and Terpenes Content

#### 2.3.1. High-Performance Liquid Chromatography-Electrospray Photo Diode Array Mass Spectrometry Analyses of LWE and Constituent Glycosides

**Figure 3:**
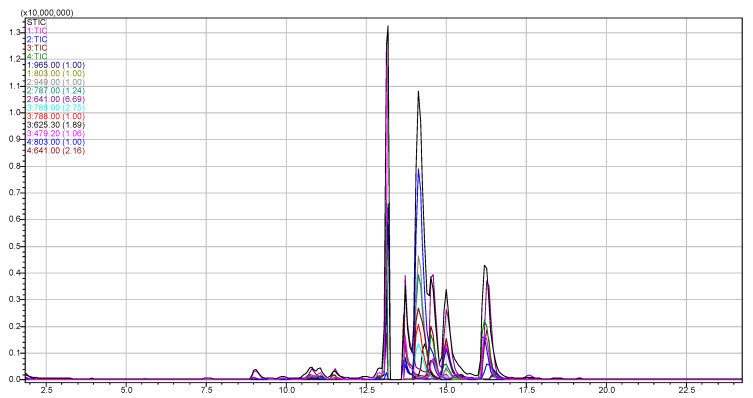
Sum and Individual Total Ion Chromatograms (TICs), and *m*/*z* ions of a diluted (100 ppm) LWE.

The four major steviol glycosides were adequately quantified while using the HPLC-PDA-ESI/MS method, both in leaves (see indicative chromatogram in Figure 3) and stems with marked concentration differences among them. Table 3 presents the analytical results. In addition, a complementary hydrophilic interaction liquid chromatographic mass spectrometric method (HILIC-PDA-ESI/MS) was concomitantly explored (see Figure S1), managing to better discriminate glycosides and resolve them in real extracts from those that share common fragment ions. Nevertheless, preliminary results showed lower sensitivity when compared to the HPLC-PDA-ESI/MS method.

#### 2.3.2. Gas Chromatography-Mass Spectrometry Analyses of EO and Constituent Terpenes

Table 4 depicts the composition of *Stevia rebaudiana* EO. Tricyclic sesquiterpene alcohol, (-)-spathulenol, caryophyllene oxide, and manool oxide were the major components.

### 2.4. Other Compounds Constituing Nematicidal Leaves Water Extract (LWE) and Wood Water Extract (WWE) Identified by Means of Ultra High Performance Liquid Chromatography Coupled to Orbitrap High Resolution Mass Spectrometry (UHPLC-HRMS)

**Figure 4:**
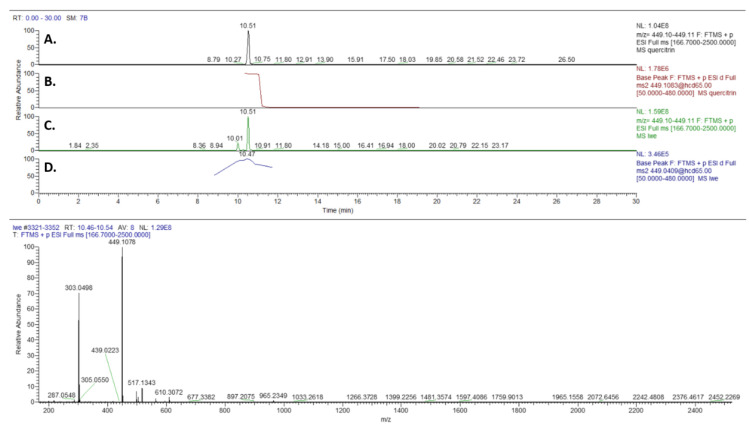
The UHPLC-HRMS chromatograms of the internal standard quercitrin (**A**) and the LWE of *S. rebaudiana* (**C**) accompanied by the respective spectrums (**B**,**D**).

Non-Targeted analysis of a leave and wood (stem) water extract was performed using UHPLC-HRMS. For the data processing procedure, the Compound Discoverer 2.1 software (Thermo Fisher Scientific, San Jose, CA, USA) was employed for retention time alignment, peak alignment, feature extraction, and detection of unknown compounds. For the annotation of the compound, the online MS^2^-library mzCloud was used. For the evaluation of the results from mzCloud library, the internal standards chlorogenic acid, rutin, quercitrin, and apigenin were included into the analysis. Table 5 presents the results after the analysis. Figure 4 presents the chromatogram and spectrum of quercitrin on LWE of *S. rebaudiana* compared to the internal standard.

## 3. Discussion

**Figure 5:**
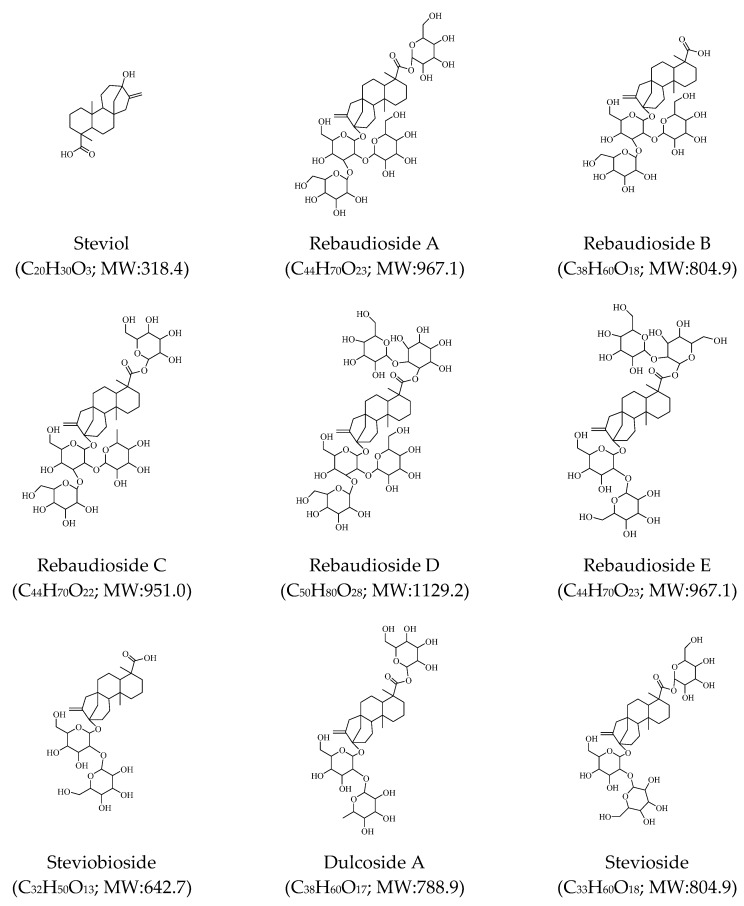
*Steviol* glycosides of the species *S. rebaudiana.*

The purification of phytochemicals and their structural elucidation of the sweet-tasting glycosides of steviol from *S. rebaudiana* leaves began approximately 90 years ago when its most abundant entkaurane glycoside, named stevioside, was extracted and crystallized [28]. Other major components whose elucidation followed were rebaudioside [29], rebaudiosides B-E, steviolbioside, and dulcoside A [30,31,32]. To date the stated specifications of the Codex Committee on Food Additives, by the Joint FAO/WHO Expert Committee on Food Additives (JECFA) of Europe, considering food products produced from *S. rebaudiana* leaves are represented by a minimum of 95% steviol glycosides. The compounds presented are stevioside, rebaudiosides A-F, rubusoside, steviolbioside, and dulcoside A [12] (Figure 5). Other than the steviol glycosides, we have identified various chemical groups of compounds, like phenols, flavonoids, terpenes, coumarin based analogues, amino and fatty acids, some of them of already proved nematicidal activity. Specifically, we previously reported the paralysis potential of terpinen-4-ol and β-caryophyllene and against *M. incognita* to exhibit an EC_50/96h_ value of 168 and 307 μg mL^−1^, respectively [33]. Interestingly, one of the major components, caryophyllene oxide, was not toxic to J2 up to the concentration of 2000 μg mL^−1^ [34], while no reports exist on the nematicidal activity of manool oxide against *Meloidogyne spp.*, Interestingly, essential oils containing (-)-spathulenol have exhibited nematicidal activity against *Meloidogyne spp.* [35,36]. To our knowledge, herein, the first report of 6-isopropenyl-4,8a-dimethyl-1,2,3,5,6,7,8,8a-octahydro-napthalen-2-ol in *S. rebaudiana* is provided. This napthalen-2-ol derivative has been reported as a constituent of the bioactive extract of *Scapania verrucose* [37], and of the bioactive root essential oil from the species *Jatropha ribifolia* [38]. According to the UHPLC-HRMS analysis of the extracts, chlorogenic acid and the flavonoids rutin, quercitrin (see its UHPLC-HRMS identification in Figure 4) and apigenin were identified while using internal standards. Additionally, several other compounds were annotated using the online library mzCloud. The majority of these constituents are largely in agreement with the ones reported in the literature on the phenolic and antioxidant compounds composition of *S. rebaudiana* [24,25,39]. To our knowledge, for some of these, it is their first putative identification in *S. rebaudiana*. More specifically, the flavonol compound, morin, and the kaempferol derivative, robinin (or kaempferol-3 *O*-robinoside-7-*O*-rhamnoside) first appear in *S. rebaudiana*. The flavonoids superfamily is known for their contribution to the chemotactic repulsion of nematodes away from the root, and their overall role in the interaction of plant-nematodes [40]. Interestingly, for the anthocyanin chloride derivative, keracyanin, it is also its first report as a constituent in *S. rebaudiana* species. Hence, this finding opens new frontiers in the chemical classes underscored in *S. rebaudiana*, unveiling new potential biosynthetic pathways in this important crop. With regard to other flavonoid counterparts, quercitrin was already isolated as one of the bioactive molecules of the aerial plant parts of *Caragana leucophloea* Pojark. (Leguminosae) [41]. Rutin was found among constituent components of the nematicidal *Croton ehrenbergii* growing unharmed amidst predators and exhibiting an innate defense mechanism against predators [42]. Quercitrin, afzelin, and quercetin were some of the phenolic components of the leaves of *Schinus terebinthifolius* significantly active against *M. incognita* [43]. Chlorogenic acid was a major component of the nematicidal water extract of *Mentha piperita*, *Mentha pulegium*, and *Mentha spicata* exhibiting significant activity against *M. incognita* [44]. Arachidonic acid is considered to be a biogenic elicitor, at concentrations of 0.1–10 μM, shown to ensure systemic, long-term protection against *M. incognita* [45]. Aqueous extracts of *Pistacia lentiscus* (L.) rich in phenolics, including quinic acid, were found active against *M. javanica* [46]. The flavonoid apigenin as isolated from the aerial parts of the species *Caragana leucophloea* Pojark. (Leguminosae) was proven to be of considerable nematicidal activity against *Caenorhabditis elegans* [41]. Last but not least, an amino acid containing plant leaf extract have exhibited nematicidal properties against *Caenorhabditis elegans* [47]. Hence, amino acids identified in this work can interplay in the demonstrated nematicidal activity.

## 4. Conclusions

According to our findings, *S. rebaudiana,* apart from being a natural non-caloric sweetener with significant medical properties for consumers, additionally exhibits noteworthy plant protection properties against the most notorious *Meloidogyne incognita* with no phytotoxicity issues on tomato host plants. The development of ecofriendly bionematicides to substitute their synthetic ancestors is now mandatory and it becomes more feasible for active plant secondary metabolites freely available in cultivated plants for food. Because both the commercialized *S. rebaudiana* leaves and the culture stem remnants were of nematicidal activity, the latter being freely available could be economically recycled into nematode control tools. We are now in the process of testing more by-products of the *S. rebaudiana* sweetener products chain, against *M. incognita*, delineating among constituents for activity. Further steps envisage the inclusion of all steviol glycosides in the respective analytical methods, alongside the in-depth exploration of bioactive compounds in *S. rebaudiana.*

## 5. Materials and Methods

### 5.1. Plant Material, Nematodes Populations and Reagents

*Stevia rebaudiana* was cultivated as an organic culture in Lamia region of Greece. The aerial parts were collected when plants had reached 10–15% οf full flowering at early July 2019 and the leaves were separated from stems (wood) and then dried in the dark at room temperature. Subsequently, they were sealed in paper bags and kept at room temperature, in the dark, until use for no longer that one month. The variety cultivated in 2019 was named SugHigh A3 and it was a kind offer of Ever Stevia, Toronto, Canada.

Nematode populations of *M. incognita* and *M. javanica* were initiated from two single eggmasses of Greek origin. They were reared on tomato plants cv. Belladonna, a variety that was susceptible to nematodes’ infestation. Freshly hatched (24 h) nematodes at the stage of second-stage juveniles (J2) were obtained according to the method of Hussey and Barker (1973) [48] from 60 day-old (d) infested roots, and were thereafter used for the experiments. 

Stevioside (98.1%), Rebaudioside A (99.5%), Rebaudioside C (99.3%), Dulcoside A (98%), and acid fuchsin were obtained from Sigma–Aldrich (Buchs, Switzerland). Water, acetonitrile, methanol and formic acid were purchased from Fisher Scientific, UK and they were of LC-MS grade. Acetone used in GC-MS analysis was of pesticide residue grade and obtained from Fisher Scientific. PTFE filters (0.45 μm) were obtained from Macherey-Nagel, Düren Germany.

### 5.2. Soil Amending with S. rebaudiana Leaves Powder (LP) and Wood Powder (WP) to Treat against M. incognita and Subsequent Biofertilization in Tomato Plants: A Dose-Response

The sandy loam soil (clay: 18%, silt: 22%, sand: 60%), with pH 6.5, 3.3% organic carbon, and 1.9 mg g^−1^ total N was collected from a noncultivated field of the Benaki Phytopathological Institute. Initially it was sieved through 3-mm and partially air dried overnight. The maximum water holding capacity and soil moisture were calculated according to Pantelelis et al., 2006 [49] and then a mixture with sand at a ratio of 2:1 was prepared to form the hereafter referred to soil. Six plastic bags represented the experimental treatments, 1kg of soil each receiving a nematode inoculation 2500 J2 kg^−1^. After appropriate mixing and overnight incubation at room temperature according to Ntalli et al., 2020 [50] the plastic bags were spiked with appropriate amounts of LP and WP to reach the test concentrations of 1, 5, 10, 50, and 100 g kg^−1^ soil. NEMguard SC (garlic extract by Intrachem) was used as a commercial control at the recommended dose (4 L ha^−1^ that is 2 μL of formulated product per kg soil or 2 mg of a.i per kg of soil) [51]. A water control was also included in the experiment. Seven-week old tomato plants, cv. Belladonna were transplanted into the treated soil, separated in five different pots containing 200g of soil each, and the bioassay was kept at 27 °C, 60% relative humidity at 16 h photoperiod for 40 days. Every pot received 20 mL of water every three days and forty days; afterwards, plants were uprooted and gently washed. Shoots were separated from roots and the latter were stained with acid fuchsin, according to Byrd et al. (1983) [52], and the following parameters were assessed: (1) *M. incognita* females per g of root at 10× magnification control, (2) fresh stems weight, and (3) fresh root weight. The experiment was performed twice, and the treatments were arranged in a completely randomized design with five replicates.

### 5.3. Essential Oil (EO) and Water Extracts (LWE & WWE) Production

The dried *S. rebaudiana* plant material was water distilled in a Clevenger apparatus (Winzer, Wertheim, Germany) for 3 h at a ratio of 1/10 (*w*/*v*) plant parts/water volume. The attained EO was dried over anhydrous Na_2_SO_4_ and was stored in dark glass vial with Teflon-sealed caps at −2.0 ± 0.5 °C until use. Before the chemical analysis, the EO was allowed to gradually reach ambient temperature. The yield of EO was determined as an average of three replicates and it was 0.004% (*w*/*w*) (data not shown).

The water extract was prepared by mixing the dried stevia leaves or wood with distilled water at a ratio of 1/10 (*w*/*v*) and then sonicated for 15 min. (Branson 1210, Marshall Scientific, Hampton, NH, USA). In extends, a filtration was performed through a Whatman no. 40 filter paper (Whatman International Ltd., Maidstone, England). All of the extracts were used fresh for bioassays. The *S. rebaudiana* yield in dry LE and WE extract were 10.8 and 10.5% (*w*/*w*), respectively, as calculated after exhaustive evaporation of the solvent (data not shown). It must be noted that this extraction method is not the one optimized for total glycosides acquirement. 

### 5.4. Paralysis Effect of Leaves Water Extract (LWE) and Wood Water Extract (WWE) on the Plant Parasitic Nematode *M. incognita* and *M. javanica* Second Stage Juveniles (J2)

The nematicidal potential of LWE and WWE, in terms of J2 paralysis, was studied, and the EC_50_ values were established. The water extract of leaves (LWE) and of wood (WWE) were both prepared by mixing the dried *S. rebaudiana* parts with distilled water at a ratio of 1/10 (*w*/*v*), sonicated for 15 min. and filtered, as stated in the previous paragraph. For each extract, a separate dose-response bioassay was performed at the range level of 5.4 to 0.042 mg mL^−1^. Crude extracts were used as stock solutions and working solutions were prepared with subsequent dilutions in distilled water. All of the test solutions were expressed per dry extract after having calculated the extract dry yield by exhaustive evaporation of the solvent (water). Distilled water served as control. Around fifteen J2 were employed per treatment well in Cellstar 96-well plates (Greiner bio-one, Frickenhausen, Germany). The plates were shielded and kept in the dark at 28 °C. Border wells were used to check the vapor drift. Juveniles were observed with the aid of an inverted microscope (Euromex, Arnhem, The Netherlands) at 40× after 1, 24, and 48 h and were separated into: motile or paralyzed. After the last assessment (48 h), the nematodes were transferred into plain water and they were evaluated again after 24 h for motility regain. Paralysis treatments were replicated six times, and every experiment was performed twice.

### 5.5. High-Performance Liquid Chromatography - Photo Diode Array Electrospray Mass Spectrometry Analyses of LWE

HPLC-DAD-ESI/MS conducted the chemical analysis of steviol glycosides. A Shimadzu (Kyoto, Japan) LCMS-2010 EV Liquid Chromatograph Mass Spectrometer instrument was used with the LCMS solution version 3.0 software consisting of an SIL-20A prominence autosampler and an SPD-M20A diode array detector. These compartments were in conjunction with a mass selective detector that was equipped with an atmospheric pressure ionization. The HPLC separation (three replicates were analyzed) was accomplished on a Zorbax Eclipse Plus, 3.5 μm, 150 × 2.6 mm i.d. chromatographic column. The mobile phase solvents composed of: (A), 0.1% formic acid in water, (B), 0.1% formic acid in acetonitrile, and (C), Methanol. The flow rate was set at 0.3 mL min.^−1^ and the mobile phase was identical with the one mentioned in the literature [53], with modification in the gradient, since not all steviol glycosides were included. The overall runtime was established at 25 min. Electron Spray Ionization (ESI) mode, using four distinct events for each analyte (see Table S1 and Figures S2 and S3), was applied in the selected ion monitoring mode (SIM). Photodiode array monitored wavelengths in the range of 190–800 nm.

### 5.6. Gas Chromatography-Mass Spectrometry Analyses of EO

The GC-MS analysis was performed on a Chromtech Evolution 3 MS/MS triple quadrupole mass spectrometer that was built on an Agilent 5975 B inert XL EI/CI MSD system that was operated in full scan data acquisition mode, covering a mass range from *m*/*z* 50 to 500. The samples were injected with a Gerstel MPS-2 autosampler using a 10-μL syringe. Separations were performed on the chromatographic column Agilent J&W HP-5ms Ultra-Inert (UI), length 30m, Inner Diameter (ID) 0.25mm, film thickness 0.25 μm (Agilent Technologies, Santa Clara, CA, USA). Helium (99.9999% purity) was used as the carrier gas at a flow rate of 1.2 mL min^−1^. The column oven temperature program started from 45 °C, staying for 1 min, increased to 250 °C at a rate of 5 °C min^−1^, where it remained for 5 min. The transfer line, manifold, and source of ionization temperatures were 300, 40 and 230 °C, respectively. The electron multiplier voltage was set at 2000 V. The total GC analysis was 47 min. (see respective chromatogram in Figure S3). The identified peaks in GC-MS (triplicate analysis) were confirmed by comparing the acquired mass spectra with those in the commercial library of NIST 08.

#### Analytical Method Validation-Quantitation of Components

The analytical method was validated consulting primarily the International Conference on Harmonization (ICH) [54], a SANTE guideline (SANTE/11813/2017) [55], and a pertinent publication [56]. The validation study concerned recovery, linearity, intra-day, and inter-day precision. Calibration curves, which were established using the dilute standard solution of the four analytes, varied from 40 to 2000 ng mL^−1^. Blank experiments were also performed, without the matrix extract. Standard addition was used for the recovery study (two concentration levels).

More specifically, the precision of the chromatographic method was expressed as the relative standard deviation values (RSD %) of the repeatability (intra-day) and intermediate precision (inter-day) analyses (*n* = 3) over one, two, and three days. Repeatability and intermediate precision were considered acceptable when RSD% were < 20%. Limit of Quantitation(s) (LoQs) were defined as the lowest validated spiked level (at 40 ng g^−1^, equivalent to 40 ng mL^−1^) that met the method performance acceptability criteria, regarding mean recoveries in the range of 70–120%, with RSDr 20% (for analytical method validation characteristics see Table S2). With regard to the Limit of Detection (LoD) values, they were defined as three times the baseline noise of the signals produced after injecting low concentration standards solutions of the four steviol glycosides mix (blank matrix is not available for stevia) (see LoD values in Table S2). Since the filtering of extracts prior to chemical analysis can affect the recovery of bioactive compounds from natural products, the filters used in this study were assessed for this purpose. The results showed a negligible effect on the recovery of the glycosides (analysis conducted before and after filtering).

To calculate matrix effects, the slopes of the calibration lines that were obtained for *S. rebaudiana* extracts after standard addition (b_matrix_) and the solvent (b_solvent_) were divided in order to determine the matrix factor and the % matrix effect (ME) was calculated by Equation (1).

%ME = (1 − b_matrix_/b_solvent_) × 100 (1)

### 5.7. Ultra High Performance Liquid Chromatpgraphy—Coupled to Orbitrap High Resolution Mass Spectrometry Analysis of Leaves Water Extract (LWE) and Wood Water Extract (WWE)

Metabolite profiling of the extracts (triplicate analysis) was performed on a Dionex Ultimate 3000 UHPLC system (Thermo Scientific™Dionex™, Sunnyvale, CA, USA) that was equipped with Q-Exactive Orbitrap mass spectrometer (Thermo Fisher Scientific, San Jose, CA, USA) on positive (ESI+) and negative (ESI-) ion mode. Chromatographic separation was achieved on a Hypersil Gold UPLC C18 (2.1 × 150 mm, 1.9 μm) reversed phased column (Thermo Fisher Scientific, San Jose, CA, USA). The mobile phases consisted of (A) ultrapure water with 0.1% formic acid and (B) acetonitrile. The elution gradient was set, as follows: 0 to 21 min: 95% A: 5% B, 21 to 24 min: 5% A: 95% B, 24 to 30 min: 95% A: 5% B. The eluent flow rate was maintained at 0.22 mL min^−1^ and the *m*/*z* ranges were set to 150–2500 Da on profile mode. HRMS operation parameters for both negative and positive modes were set as follows: capillary temperature, 350 °C; spray voltage, 2.7 kV; S-lense Rf level, 50 V; sheath gas flow, 40 arb. units; aux gas flow, 5 arb. units; aux. gas heater temperature, 50 °C. Each sample was analyzed in full scan mode at a resolving power of 70,000, whereas, for the data dependent acquisition mode, the resolution was 35,000 allowing for MS/MS fragmentation of the three most intense ions. Stepped normalized collision energy was set at 35, 60, and 100. The column temperature was kept throughout the analysis at 40 °C, while the sample tray temperature was set at 4 °C. A 5 µL aliquot of each sample was injected. Data analysis was achieved using Compound Discoverer 2.1 software (Thermo Fisher Scientific, San Jose, CA, USA) for alignment, peak peaking, and grouping. For metabolite annotation, the online MS^2^-library mzCloud was used applying *m*/*z* tolerance of 5 ppm and taking into consideration the isotopic and MS/MS fragmentation pattern.

### 5.8. Statistics

Natural death/paralysis was eradicated according to the Schneider Orelli formula [57], which is corrected % = [(mortality% in treatment − mortality % in control)/(100 − mortality % in control)}] × 100, and experiments analyzed (ANOVA) were combined over time. The means were averaged over bioassays since ANOVA showed no significant treatment by time interaction. Corrected percentages of paralyzed J2 were subjected to nonlinear regression analysis using the log-logistic equation proposed by Seefeldt et al. [58]: Y = C þ (D − C)/{1 þ exp[b (log(x) − log(EC50))]}, where C = the lower limit, D = the upper limit, b = the slope at the EC_50_, and EC_50_ = the test concentration required for 50% death/paralysis after removal of the control (natural death/paralysis). In the regression equation, the test concentration was the independent variable (x) and the paralyzed J2 (percentage increase over water control) was the dependent variable (y). The mean value of the six replicates per test concentration and immersion period was used to calculate the EC_50_ value. The 95% confidence intervals (95% CI) were determined for toxicity comparison.

The means were averaged over bioassays when considering pot bioassays and since ANOVAs showed no significant treatment between runs of experiment. The data from the pot bioassays were expressed as a percentage decrease in the number of females per g of root corrected according to the control (water), using the Abbott’s formula: corrected % = 100 × {1 − [females number in treated plot/females number in control plot]}. It was fitted in the log-logistic model, as for paralysis data, in order to estimate the concentration that caused a 50% decrease in females per g of root (EC_50_ value). The 95% confidence intervals (95% CI) were determined for toxicity comparison.

## Figures and Tables

**Table 1:** Efficacy results expressed as EC_50_ (g kr^−1^) values of *M. incognita* calculated after incorporation of (A) leaves powder (LP) and (B) wood powder (WP) in pots hosting tomato plants artificially inoculated with nematodes. (–) not calculated.

EC_50_ (g kg^−1^) (Abbott: ♀ g^−1^ Root)	Std. Error	CI_95_%
*LP*		
<1	-	-
*WP*		
3.13	0.564	1.96–4.29

**Table 2:** Efficacy results expressed as EC_50_ (% w/v) values of *M. incognita* and *M. javanica* after immersion of J2s in test solutions of Leaves Water Extract (LWE) and Wood Water Extract (WWE) for 1, 24, and 48 h. (–) not calculated.

	Exposure	EC_50_ (mg mL^−1^)	Std. Error	CI_95_%
	*LWE*			
*M. incognita*	1 h	2.86	0.39	2.04–3.68
	24 h	2.30	0.29	1.70–2.90
	48 h	1.36	0.17	1.00–1.73
*M. javanica*	1 h	3.11	0.33	2.43–3.80
	24 h	0.41	0.04	0.32–0.49
	48 h	0.68	0.09	0.49–0.88
	*WWE*			
*M. incognita*	1 h	>5.4	-	-
	24 h	0.30	0.02	0.25–0.34
	48 h	0.95	0.07	0.79–1.10
*M. javanica*	1 h	>5.4	-	-
	24 h	0.42	0.04	0.34–0.51
	48 h	0.51	0.05	0.39–0.63

**Table 3:** Quantitative results (*n* = 3) for selected-key steviol glycosides determined in *Stevia rebaudiana* leaves and stems.

	*S. rebaudiana* Leaves	*S. rebaudiana* Stems
Constituent	Concentration (mg g^−1^) *	
Rebaudioside A	21.468 ± 0.181	3.937 ± 0.045
Rebaudioside C	9.679 ± 0.220	0.643 ± 0.037
Dulcoside A	1.076 ± 0.047	0.076 ± 0.003
Stevioside	19.729 ± 0.135	3.625 ± 0.064

* mg g^−1^ dried leaves or stems.

**Table 4:** Chemical and relative composition of essential oil of *Stevia rebaudiana.*

Analyte	Retention Time (min)	RI *	Relative Amount (%)
α-Terpineol	14.56	1189 (1189)	0.47 ± 0.08
α-Bourbonene	19.97	1384 (1384)	0.15 ± 0.04
β-Maaliene	20.64	1405 (1415)	0.09 ± 0.04
Caryophyllene	20.92	1419 (1417)	0.32 ± 0.06
Aromadendrene	21.32	1440 (1439)	0.67 ± 0.08
epi-β-Caryophyllene	21.86	1466 (1465)	1.02 ± 0.11
β-Guaiene	22.00	1490 (1490)	0.77 ± 0.09
β-Ionone	22.62	1491 (1490)	3.11 ± 0.21
Eremophilene	22.97	1499 (1502)	1.02 ± 0.15
γ-Cadinene	23.42	1513 (1511)	1.24 ± 0.09
(-)-β-Cadinene	23.63	1518 (1518)	1.33 ± 0.23
Cadala-1(10),3,8-triene	24.14	1555 (1562)	0.36 ± 0.05
Nerolidol	24.60	1564 (1565)	2.83 ± 0.33
(-)-Spathulenol	25.08	1577 (1578)	22.81 ± 1.49
Caryophyllene oxide	25.19	1581 (1582)	20.18 ± 1.15
Isoaromadendrene epoxide	25.81	1589 (1594)	4.24 ± 0.41
t-Cadinol	26.55	1640 (1639)	5.88 ± 0.52
α-Cadinol	26.87	1653 (1650)	3.82 ± 0.27
6-Isopropenyl-4,8a-dimethyl-1,2,3,5,6,7,8,8a-octahydro-napthalen-2-ol	27.30	1690 (1690)	3.10 ± 0.28
Ent-Germacra-4(15),5,10,(14)-trien-1β-ol	27.66	1695 (1694.5)	0.73 ± 0.10
*Unidentified*	32.97	-	1.92 ± 0.15
*Unidentified*	33.24	-	3.03 ± 0.30
*Unidentified*	33.60	-	1.63 ± 0.29
Manool oxide	34.3	1992 (1989)	17.19 ± 1.02
Epimanoyl oxide	34.76	2011 (2010)	1.74 ± 0.27
Oxomanoyl oxide	38.66	2207 (2208)	0.35 ± 0.10

* RI, retention index on HP5-MS UI column (relative to *n*-alkanes), identification based on mass spectra comparison with the reference databases, and comparison with literature RIs (depicted in parentheses).

**Table 5:** Identification and potential annotation of compounds from leaves water extract (LWE) and wood water extract (WWE) by means of Ultra High Performance Liquid Chromatography coupled to Orbitrap High Resolution Mass Spectrometry (UHPLC-HRMS) analysis.

*m/z*	t_R_^1^ (min)	Molecular Formula	Adduct	Dppm ^2^	Identification/Annotation	WWE	LWE
355.1023	8.09	C_16_H_18_O_9_	[M+H]^+^	−0.56	Chlorogenic acid *	+	+
611.1611	9.60	C_27_H_30_O_16_	[M+H]^+^	−1.15	Rutin *	+	+
449.1078	10.52	C_21_H_20_O_11_	[M+H]^+^	0.22	Quercitrin *	+	+
271.0602	13.06	C_15_H_10_O_5_	[M+H]^+^	0	Apigenin *	-	+
353.0879	8.07	C_16_H_18_O_9_	[M-H]^-^	3.40	Neochlorogenic acid	+	+
447.0935	10.52	C_21_H_20_O_11_	[M-H]^-^	2.91	Astragalin	+	+
433.1132	11.12	C_21_H_20_O_10_	[M+H]^+^	0.69	Afzelin	+	+
303.0500	10.53	C_15_H_10_O_7_	[M+H]^+^	0.33	Quercetin	+	+
305.2478	18.44	C_20_H_32_O_2_	[M+H]^+^	0.98	Arachidonic acid	+	+
193.0709	1.80	C_7_H_12_O_6_	[M+H]^+^	1.04	Quinic acid	+	+
191.0190	2.38	C_6_H_8_O_7_	[M-H]^-^	2.09	Citric acid	+	+
611.1611	9.60	C_27_H_30_O_16_	[M+H]^+^	0.65	Luteolin-3’,7-Diglucoside	+	+
303.0497	10.51	C_15_H_10_O_7_	[M+H]^+^	−0.66	Morin	-	+
433.1129	10.65	C_21_H_20_O_10_	[M+H]^+^	0	Apigetrin	+	+
595.1660	9.95	C_27_H_30_O_15_	[M+H]^+^	0.50	Keracyanin	-	+
435.0925	10.28	C_20_H_18_O_11_	[M+H]^+^	0.69	Avicularin	+	+
375.1077	15.26	C_19_H_18_O_8_	[M+H]^+^	0.80	5,2’-Dihydroxy-6,7,8,6’-tetramethoxyflavone	-	+
465.1031	9.85	C_21_H_20_O_12_	[M+H]^+^	0.86	Quercetin-3β-D-glucoside	+	+
741.2245	9.45	C_33_H_40_O_19_	[M+H]^+^	1.08	Robinin	-	+
517.1344	10.43	C_25_H_24_O_12_	[M+H]^+^	0.58	4,5-Dicaffeoylquinic acid	+	+
341.0866	7.39	C_15_H_16_O_9_	[M+H]^+^	−0.29	Esculin	-	+
359.1490	9.69	C_20_H_22_O_6_	[M+H]^+^	0.28	Matairesinol	-	+
285.2214	15.89	C_20_H_28_O	[M+H]^+^	0.35	(9cis)-Retinal	-	+
175.1190	1.69	C_6_H_14_N_4_O_2_	[M+H]^+^	0	DL-Arginine	+	+
277.1393	1.69	C_11_H_20_N_2_O_6_	[M+H]^+^	−0.36	L-Saccharopine	-	+
182.0813	2.38	C_9_H_11_NO_3_	[M+H]^+^	0.55	L-Tyrosine	+	+
303.2319	15.89	C_20_H_30_O_2_	[M+H]^+^	0	Eicosapentaenoic acid	-	+
295.2269	19.53	C_18_H_30_O_3_	[M+H]^+^	0.34	9-Oxo-10(E),12(E)-octadecadienoic acid	-	+
321.2425	15.88	C_20_H_32_O_3_	[M+H]^+^	0.31	(3S)-5-[(4aR,8aS)-2,5,5,8a-Tetramethyl-3-oxo-4a,6,7,8-tetrahydro-4H-naphthalen-1-yl]-3-methylpentanoic acid	-	+
268.1041	2.37	C_10_H_13_N_5_O_4_	[M+H]^+^	0.37	Adenosine	+	-

* The compounds chlorogenic acid, rutin, quercitrin and apigenin were identified using internal standards by comparing the accurate mass, the retention time and the MS/MS fragmentation pattern. The rest of compounds were annotated based on the mzCloud library. ^1^. t_R_, retention time, ^2^. Dppm, mass error in ppm.

## Supplementary Materials

The following are available online at https://www.mdpi.com/2072-6651/12/5/319/s1, Table S1. Characterization of steviol glycosides by HPLC-DAD-ESI/MS, Table S2. HPLC-PDA-ESI/MS Analytical Method Validation Characteristics, Figure S1. HILIC-PDA-ESI/MS TIC chromatogram (sum and separate TICs) of a standard mix solution (at 2 μg mL^−1^) of the four glycosides monitored, Figure S2. Sum and individual total ion chromatograms (TIC) (including *m*/*z* ions) of a standard mix solution (at 2 μg mL^−1^) of the four glycosides monitored, Figure S3. Rebaudioside A detection in the methanolic extract of Stevia leaves (SIM chromatogram).

## Abbreviations

LP	Culture residues in the form of leaves powder
WP	culture residues in the form of wood powder
LWE	leaves water extract
WWE	wood water extract
EO	essential oil
GC/MS	Gas Chromatography/Mass Spectrometry
UHPLC-HRMS	Ultra High Performance Liquid Chromatography coupled to Orbitrap High Resolution Mass Spectrometry
HPLC-PDA-ESI/MS	High-Performance Liquid Chromatography-Photo Diode Array Electrospray Mass Spectrometry
