# Supplementary material for: Nematicidal Activity of Stevia rebaudiana (Bertoni) Assisted by Phytochemical Analysis
Source: Toxins (Basel). 2020 May 12;12(5):319. doi: 10.3390/toxins12050319 (PMC7290675; doi:10.3390/toxins12050319)
Supplement: Supplementary file 1 [file toxins-12-00319-s001.pdf]

Supplementary material: Nematicidal Activity of *Stevia rebaudiana* (Bertoni) assisted by Phytochemical Analysis

Nikoletta Ntalli, Konstantinos M. Kasiotis, Eirini Baira, Christos L. Stamatis and Kyriaki Machera

Table S1. Characterization of steviol glycosides by HPLC-DAD-ESI/MS.

Compound Name.	t_R (min)	Quantitation ion	Confirmation ion(s)	Mode polarity	Voltage (kV)	Event N°
Rebaudioside A	13.27	965	803	ESI(-)	2	Event 1
Rebaudioside C	14.21	949	787, 641	ESI(-)	1.9	Event 2
Dulcoside A	14.33	625.3	788	ESI(-)	2	Event 3
Stevioside	13.26	803	641, 479.2	ESI(-)	2	Event 4

Table S2. HPLC-PDA-ESI/MS Analytical Method Validation Characteristics.

					Recovery ±RSD % n = 3		Inter-d precision (RSD % n = 3)	Intra-d-precision (RSD % n = 3)
**Analyte**	**Regression Equation***	**Regression coefficient (R^2)**	**LoD (ng mL^{-1})****	**ME (%)*****	40 ng g^{-1}	400 ng g^{-1}	40 ng g^{-1}	40 ng g^{-1}
Rebaudioside A	y=180.78x+3742.1	0.9988	9	-7.8	77±8	83±9	5.46	7.02
Rebaudioside C	y=105.02x+702.77	0.9999	12	-5.9	90±9	93±14	1.48	1.22
Dulcoside A	y=199.79x-1545.2	0.9991	9	-1.8	83±8	82±10	4.71	2.79
Stevioside	y=226.25x+1518.8	0.9997	9	-7.3	81±7	90±13	4.16	4.02

* Calibration Range 40-2000 (ng/mL), ** LoD, Limit of Detection, *** ME: Matrix Effect.

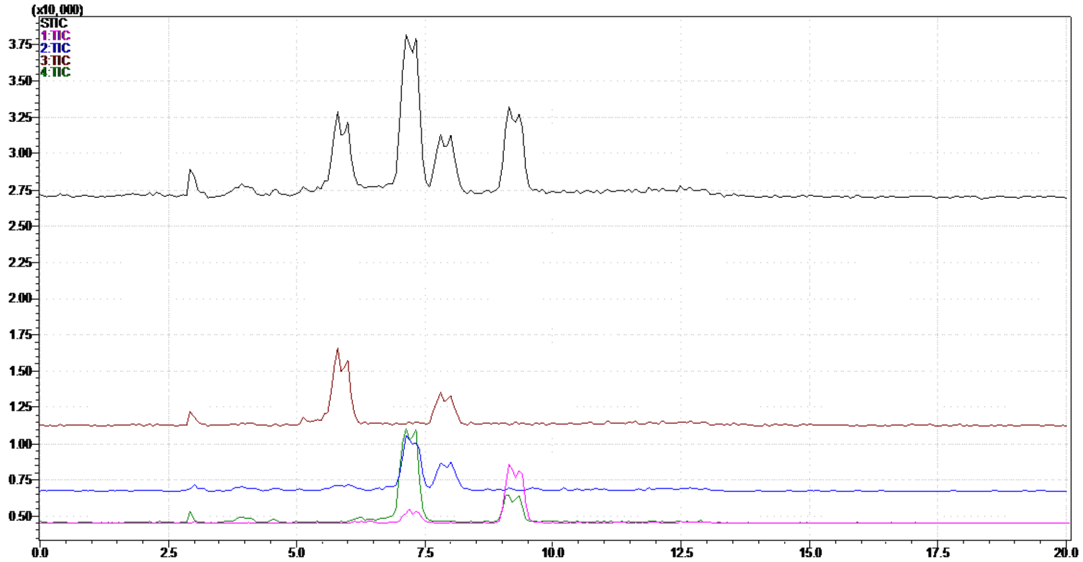

Figure S1. HILIC-PDA-ESI/MS TIC chromatogram (sum and separate TICs) of a standard mix solution (at 2 μg mL^{-1}) of the four glycosides monitored.

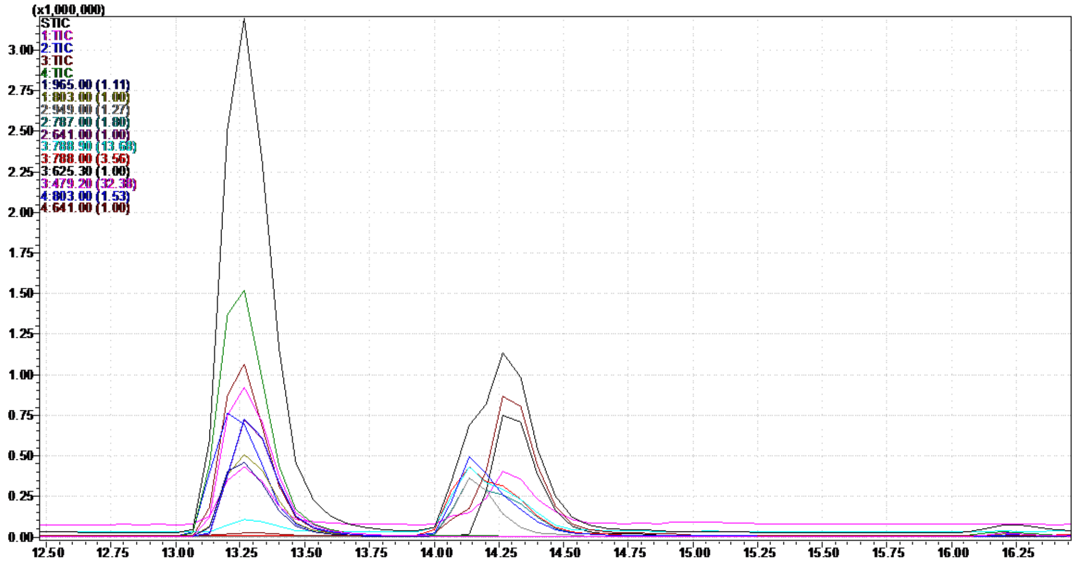

Figure S2. Sum and individual total ion chromatograms (TIC) (including *m*/*z* ions) of a standard mix solution (at 2 μg mL^{-1}) of the four glycosides monitored.

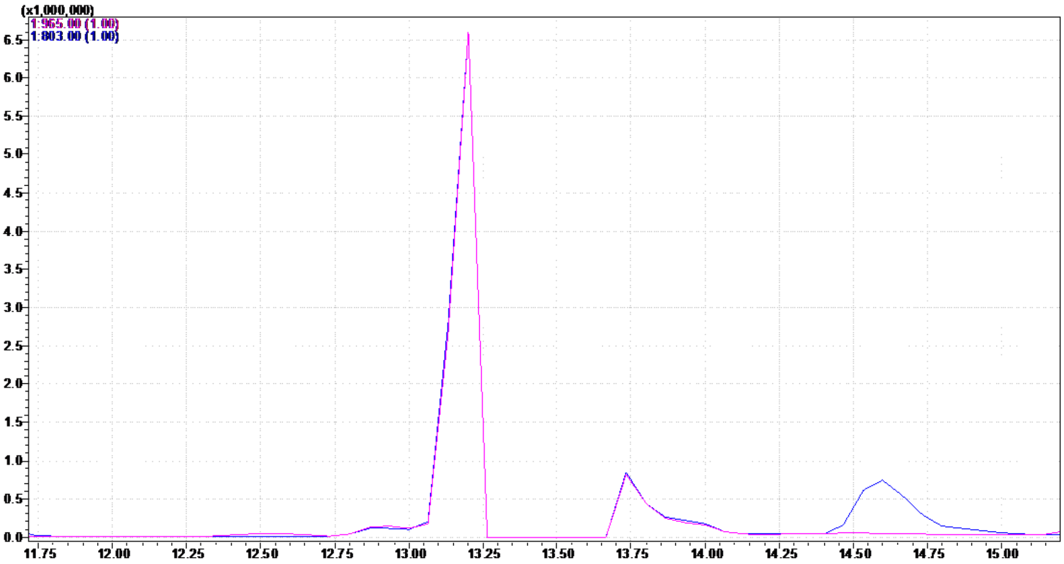

Figure S3. Rebaudioside A detection in the methanolic extract of Stevia leaves (SIM chromatogram).
